# Beam collimation and filtration optimization for a novel orthovoltage radiotherapy system

**DOI:** 10.1002/mp.17662

**Published:** 2025-02-06

**Authors:** Nathan Clements, Olivia Masella, Deae‐Eddine Krim, Lane Braun, Magdalena Bazalova‐Carter

**Affiliations:** ^1^ Department of Physics and Astronomy University of Victoria Victoria British Columbia Canada; ^2^ Department of Mechanical Engineering University of Victoria Victoria British Columbia Canada

**Keywords:** bayesian optimization, collimator, kilovoltage radiotherapy, monte carlo simulation, optimization, orthovoltage, radiation therapy

## Abstract

**Background:**

The inaccessibility of clinical linear accelerators in low‐ and middle‐income countries creates a need for low‐cost alternatives. Kilovoltage (kV) x‐ray tubes have shown promise as a source that could meet this need. However, performing radiotherapy with a kV x‐ray tube has numerous difficulties, including high skin dose, rapid dose fall‐off, and low dose rates. These limitations create a need for highly effective beam collimation and filtration.

**Purpose:**

To improve the treatment potential of a novel kV x‐ray system by optimizing an iris collimator and beam filtration using Bayesian techniques and Monte Carlo (MC) simulations.

**Methods:**

The Kilovoltage Optimized AcceLerated Adaptive therapy system's current beam configuration consists of a 225 kVp x‐ray tube, a 12‐leaflet tungsten iris collimator, and a 0.1 mm copper filter. A Bayesian optimization was performed for the large and small focal spot sizes of the kV x‐ray tube source at 220 kVp using TopasOpt, an open‐source library for optimization in TOPAS. Collimator thickness, copper filter thickness, source‐to‐collimator distance (SCD), and source‐to‐surface distance (SSD) were the variables considered in the optimization. The objective function was designed to maximize the dose rate and the dose at a depth of 5 cm while minimizing the beam penumbra width and the out‐of‐field dose (OFD), all evaluated in a water phantom. Post‐optimization, the optimal beam configuration was simulated and compared to the existing configuration.

**Results:**

The optimal collimation setup consisted of 2.5 mm thick tungsten leaflets for the iris collimator and a 350 mm SSD for both focal spot sizes. The optimal copper filtration was 0.22 mm for the large focal spot and 0.15 mm for the small focal spot, with a SCD of 148.5 mm for the large focal spot and 125.8 mm for the small focal spot. For the large focal spot, the surface dose rate decreased by 9.4%, while the PDD at 5cm depth (${\text{PDD}}_{5cm}$) increased by 7.7% compared to the existing iris collimator. Additionally, the surface beam penumbra width was reduced by 31.3%, and no significant changes in the OFD were observed. For the small focal spot, the surface dose rate for the new collimator increased by 3.7% and the ${\text{PDD}}_{5cm}$ increased by 5.3%, with no statistically significant changes in the beam penumbra width or OFD.

**Conclusion:**

The optimal beam collimation and filtration for both x‐ray tube focal spot sizes of a kV radiotherapy system was determined using Bayesian optimization and MC simulations and resulted in improved dose distributions.

## 1 INTRODUCTION

In modern radiation therapy (RT), research is often focused on new advanced technologies or methodologies which provide relatively small improvements in cancer treatment in first‐world nations. However, minimal effort is put into providing alternatives to the expensive linear accelerators (linacs) that are not available or affordable to a large global population in lower‐ and middle‐income countries or even in remote regions of some first‐world nations such as Canada and Australia.^1^, ^2^ One potential treatment alternative would be to employ kV x‐ray tubes as radiation sources, given their relative inexpensiveness compared to clinical megavoltage (MV) linacs.

Compared to MV photon beam treatments, kV RT has several benefits and drawbacks. In particular, the drawbacks of kV x‐rays are: substantial skin dose (∼100% of $D_{max}$ for 200 kV), a rapid dose fall‐off (∼20% of $D_{max}$ at 10cm depth for 200 kV), and long treatment times for deep‐seated tumors (∼20min).^3^ However, kV x‐ray treatments do benefit from a short secondary electron range (1–2 mm) which reduces the dose spread from the target, from minimal shielding requirements (2‐4mm of lead), and a low cost as a result of inexpensive shielding and equipment (∼200k USD).^3^ Presently, 50‐300 kV x‐rays are used only for superficial cancer treatment and intraoperative RT (IORT) due to the high surface dose and rapid dose fall‐off.^4^, ^5^, ^6^ Nevertheless, recent simulation studies have shown promise for kilovoltage x‐ray arc therapy. O'Connell et al. recently showed that simulated non‐coplanar, isocentric lung treatments using kV beam arcs complied with all Radiation Therapy Oncology Group (RTOG) 0813 dosimetric criteria for two patient cases.^7^ A similar simulation study from 2019 showed that kilovoltage x‐ray arc therapy plans were within the constraints set by the American Association of Physicists in Medicine Task Group 101 for 2/3 of the considered lung patient cases.^8^

Beam collimation is a crucial aspect of RT, designed to shape and direct the treatment beam to the target while minimizing radiation dose to surrounding healthy tissues. Collimators, typically made of high‐density and high atomic number materials such as tungsten or lead, restrict the size and shape of the radiation beam to conform closely to the tumor volume. This reduces the dose to adjacent organs and tissues, improving the therapeutic ratio of treatment. Modern RT systems utilize multi‐leaf collimators (MLCs) that allow for dynamic adjustments during treatment, enabling complex shapes and intensity‐modulated radiotherapy (IMRT).^9^ Effective collimation enhances treatment accuracy and reduces side effects by sharpening the beam penumbra and limiting out‐of‐field dose (OFD), making it a vital aspect of treatment delivery.

**FIGURE 1:**
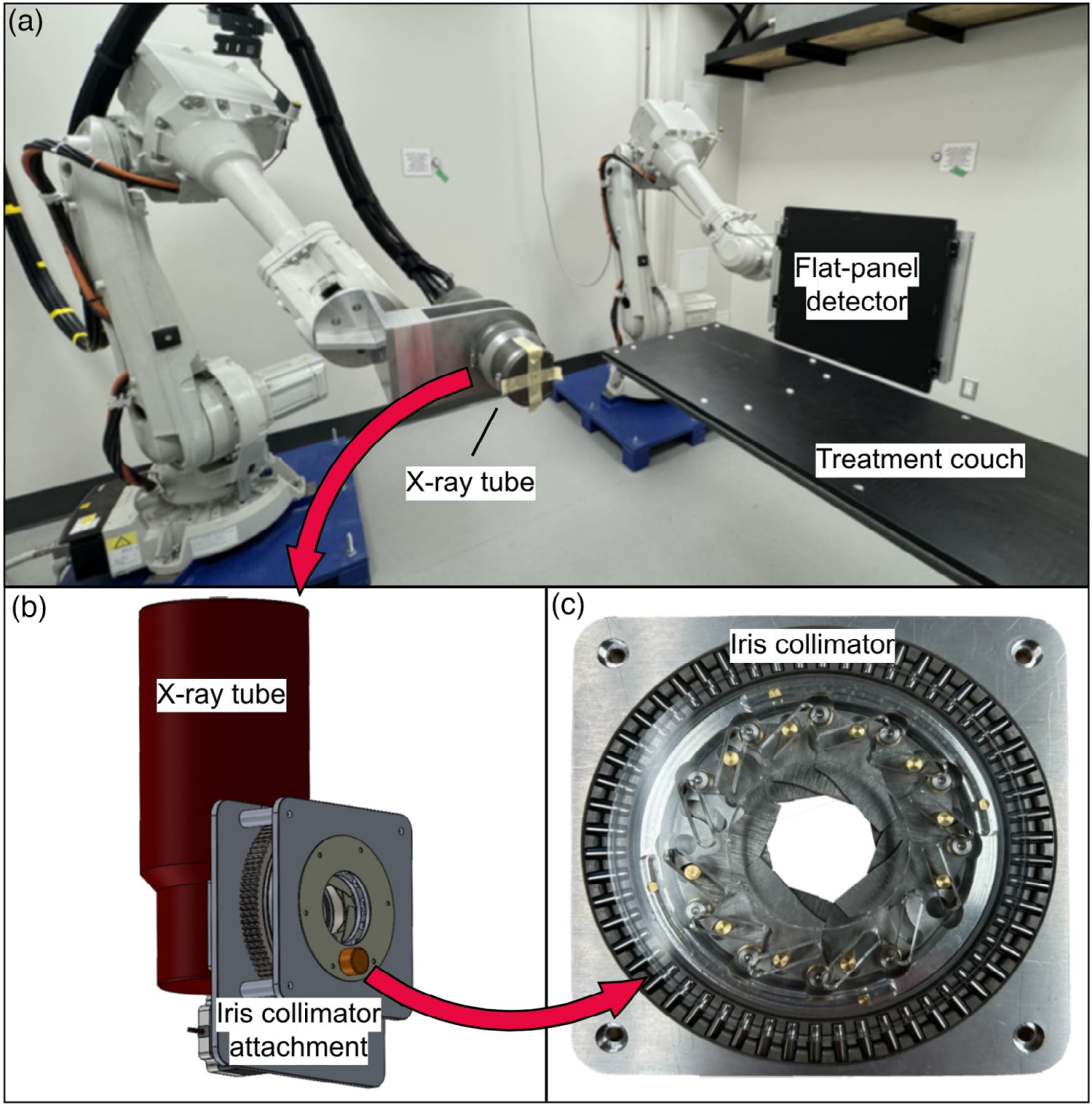
(a) A photo of the KOALA system. (b) A 3D model of the x‐ray tube and collimation. (c) A photo of the 12‐leaflet tungsten iris collimator. KOALA, Kilovoltage Optimized AcceLerated Adaptive therapy.

The Kilovoltage Optimized AcceLerated Adaptive therapy (KOALA), Figure 1a system is a novel orthovoltage treatment system being developed at the University of Victoria. The system is designed to be low‐cost with the ultimate goal of being accessible to low‐ and middle‐income countries and remote areas with no access to conventional clinical linear accelerators. The system consists of two IRB4600 robotic arms (ABB, Zurich, Switzerland) one that holds a Comet MXR‐225/26 x‐ray tube (Flamatt, Switzerland) with a nominal tube voltage of 225 kV, and one that holds a 17″ × 17″ Mercu1717V flat panel a‐Si detector (iRAy Technology, Shanghai, China). The system is capable of cone beam CT imaging and is being developed to perform radiotherapy treatments of canine patients using non‐coplanar arcs. The collimation system considered in this work consists primarily of a twelve‐leaflet tungsten iris collimator which has recently been developed and is an inexpensive and simpler collimation system compared to MLCs. Similarly, a team at Stanford has employed an iris collimator to adapt a kV micro‐CT small‐animal platform for conformal radiotherapy.^10^, ^11^

Bayesian optimization works by fitting a surrogate function to the unknown objective function and extracting values of the true objective function to continuously update and improve the modeled objective function.^12^ The surrogate function used in this work was a Gaussian process model. Gaussian processes can be understood as infinite‐dimensional generalizations of multivariate Gaussian distributions. The optimizer calculates the objective function value at each iteration and adjusts the model accordingly, gradually guiding it toward convergence with the true objective function. Bayesian optimization has for example been used for the optimization and design of a novel x‐ray shaping device as an alternative to multi‐leaf collimation,^13^ optimization of a laser‐Compton x‐ray source for imaging,^14^ and several treatment planning studies including proton therapy,^15^ IMRT,^16^ and brachytherapy.^17^ To improve the treatment capability of the KOALA system, a Bayesian optimization of the beam collimation and filtration variables was performed.

This work used Monte Carlo (MC) simulations to perform a Bayesian optimization of beam collimation and filtration of the KOALA kV x‐ray RT system. The optimization variables included collimator thickness, copper filter thickness, source‐to‐collimator distance (SCD), and source‐to‐surface distance (SSD). These variables were optimized by maximizing dose rate and depth dose while minimizing beam penumbra width and OFD to improve treatment delivery.

## 2 METHODS

### 2.1 The current beam collimation and filtration configuration

The current collimator is a dodecagonal iris collimator (Figure 1c) that attaches directly to the x‐ray tube (Figure 1b) held by one of the robot arms. The iris collimator consists of twelve 1 mm thick tungsten leaflets. Depending on the iris collimator opening, the degree of leaf overlap varies, which results in different parts of the beam potentially passing through one, two, or even three leaflets. The collimator has an inherent SCD from the x‐ray tube focal spot to the upstream face of the leaflets of 102 mm and contains a filter holder at 51 mm from the focal spot. In its current configuration, the KOALA x‐ray system uses 0.1 mm of copper for beam filtration and a source‐to‐isocenter distance of 40 cm. The beam collimation and optimization process is described below.

### 2.2 MC simulation and optimization software

TOPAS v3.9^18^, ^19^ was used to run all MC simulations to calculate dose from the KOALA system in a water phantom. The Bayesian optimizer developed in TopasOpt,^20^ an open‐source library for performing mathematical optimization of parameters was used for the optimization.

### 2.3 MC simulations of the current beam collimation and filtration

**FIGURE 2:**
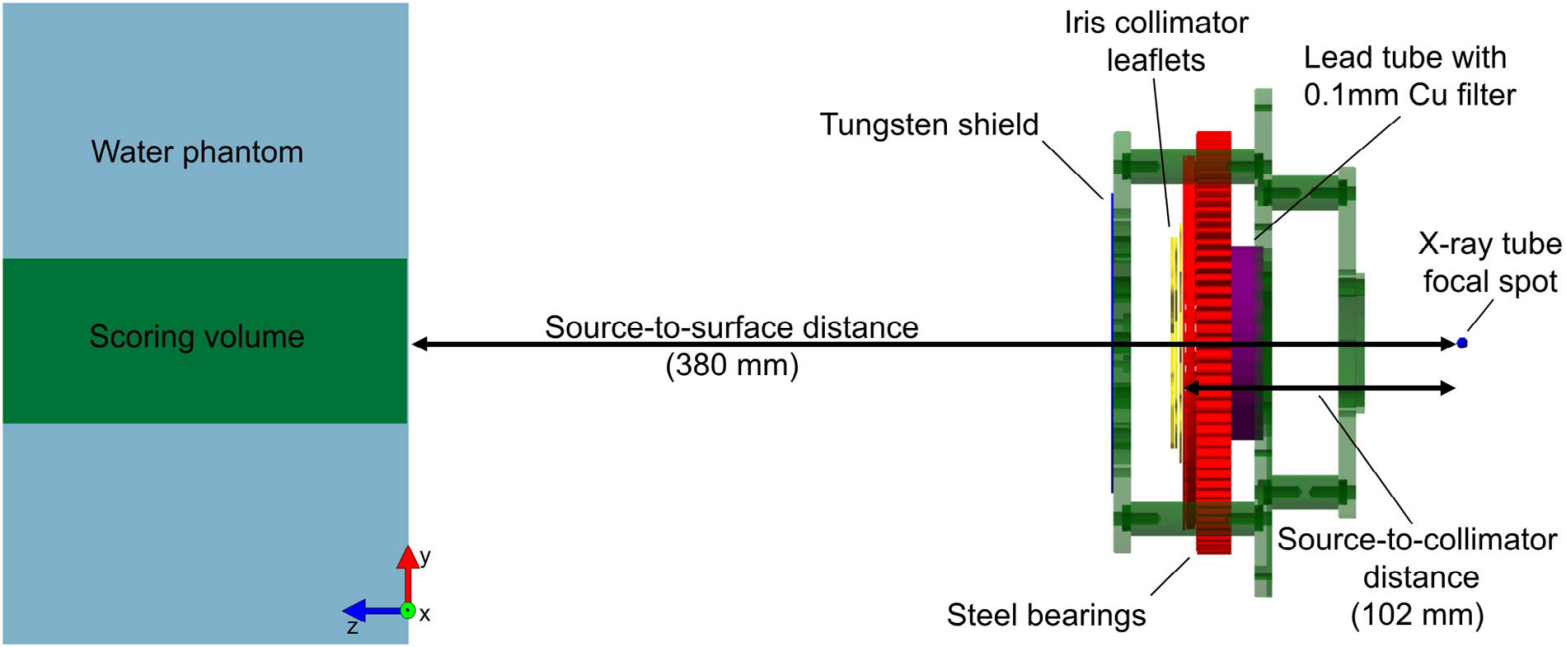
MC simulation setup for water phantom dose calculations using the existing iris collimator. MC, Monte Carlo.

The current x‐ray beam was simulated using TOPAS MC, as shown in Figure 2. The simulation setup consisted of the iris collimator with 1 mm thick tungsten leaflets set to produce a 3 cm field size at isocenter, a downstream tungsten shield, the steel bearings which move with the leaflets, a lead tube, all simulated as .stl files. Additionally, a 0.1 mm copper filter, and a (30 × 30 × 20) ${\text{cm}}^{3}$ water phantom were simulated, with the surface of the phantom at 38 cm from the x‐ray tube focal spot (38 cm SSD), such that the isocenter was at 2‐cm depth within the phantom. The sources were phase‐space sources generated using EGSnrc/BEAMnrc code,^21^ to simulate the x‐ray tube using the two circular focal spots with diameters of 1.2 mm (small) and 5.5 mm (large). A 220keV monoenergetic beam of electrons with a rectangular shape to mimic the focal spot size was simulated incident on a tungsten anode, angled 30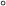, using the XTUBE module. The 2mm beryllium window and exit aperture were similarly simulated to improve the accuracy of the model. Directional bremsstrahlung splitting was used as a variance reduction technique with a splitting number of 500 and a splitting field located at the exit aperture of the x‐ray tube. For each focal spot, $10^{9}$ electrons were simulated, resulting in approximately $2.2\times 10^{8}$ photons scored. EGSnrc/BEAMnrc was chosen over TOPAS to simulate the x‐ray tube due to its greater calculation efficiency.^22^ Mean dose and standard deviation were scored in the water phantom with a (60 × 60 × 200) ${\text{mm}}^{3}$ scoring volume in (1 × 1 × 5) ${\text{mm}}^{3}$ voxels shown in Figure 2. MC uncertainty was <1% for the calculated quantities.

The physics module used was the “g4em‐standard‐opt4” package which has the most accurate electromagnetic interactions module and settings. A cutoff distance of 5 μm was employed and all other physics parameters were kept as their default. Simulations were run on the Digital Research Alliance of Canada's NARVAL computer cluster using multi‐threading across 64 CPUs. The large and small focal spot simulations each took approximately 44 h.

### 2.4 Bayesian optimization of beam collimation and filtration

To determine the optimal collimation and filtration, Bayesian optimization with Gaussian processes was employed using TopasOpt.^20^ The thickness of the tungsten collimator, the thickness of the copper filter, the SCD, and the SSD were optimized. It is important to note that for the optimization, the iris collimator was approximated as a block of tungsten with a parallel circular hole which created a 3cm beam size at the isocenter. This was done since for each iteration of the optimizer with a changed collimator thickness, SSD, or SCD, a new iris collimator .stl file would have had to be produced to maintain the correct beam size at the isocenter. The hole size was always such that a 3cm beam would be achieved at the 2 cm depth isocenter. The range for each variable considered in the optimization was selected based on the geometric constraints of the system and practical insights from prior experiments and usage. The existing collimator and filtration values and the ranges considered for the optimization process are presented in Table 1.

**TABLE 1:** The existing values for the collimator and filtration variables and the ranges considered for the optimization.

Collimation and filtration variables	Existing value	Optimization range
Collimator thickness	1 mm leaflets	1–10 mm^a^
Copper filter thickness	0.1 mm	0.05–1 mm
SCD	102 mm	50–200 mm
SSD	380 mm	350–500 mm

^a^ For the optimization the collimator was simulated as a simple block with a hole rather than the iris.

Abbreviations: SCD, source‐to‐collimator distance; SSD, source‐to‐surface distance.

The terms of the objective function were selected to enhance the treatment capabilities of the system. Two parameters were to be maximized: (1) the dose rate, to shorten treatment times and increase patient throughput, and (2) the dose at depth, to improve the system's potential for treating deep‐seated targets. Two other parameters were to be minimized: (1) beam penumbra width, to improve dose conformity to the treatment volume; and (2) OFD, to reduce radiation exposure to surrounding healthy tissues. To maximize the efficiency of the optimization, the objective function parameters were calculated at surface, allowing for lower particle source statistics and smaller scoring volumes, reducing simulation time. However, to consider dose at depth, a second scoring volume was used so that the central axis (CAX) percentage depth dose at 5cm depth in water (${\text{PDD}}_{5cm}$) could be evaluated.

Similar to Section 2.3, the optimization was performed for both the large and small focal spots and used the same phase spaces as described there. However, only $75\times 10^{6}$ of the $10^{9}$ original histories were run to improve simulation time, each $75\times 10^{6}$ history simulation/iteration of the optimizer took around 35 min. MC uncertainty was <3% for the calculated quantities.

**FIGURE 3:**
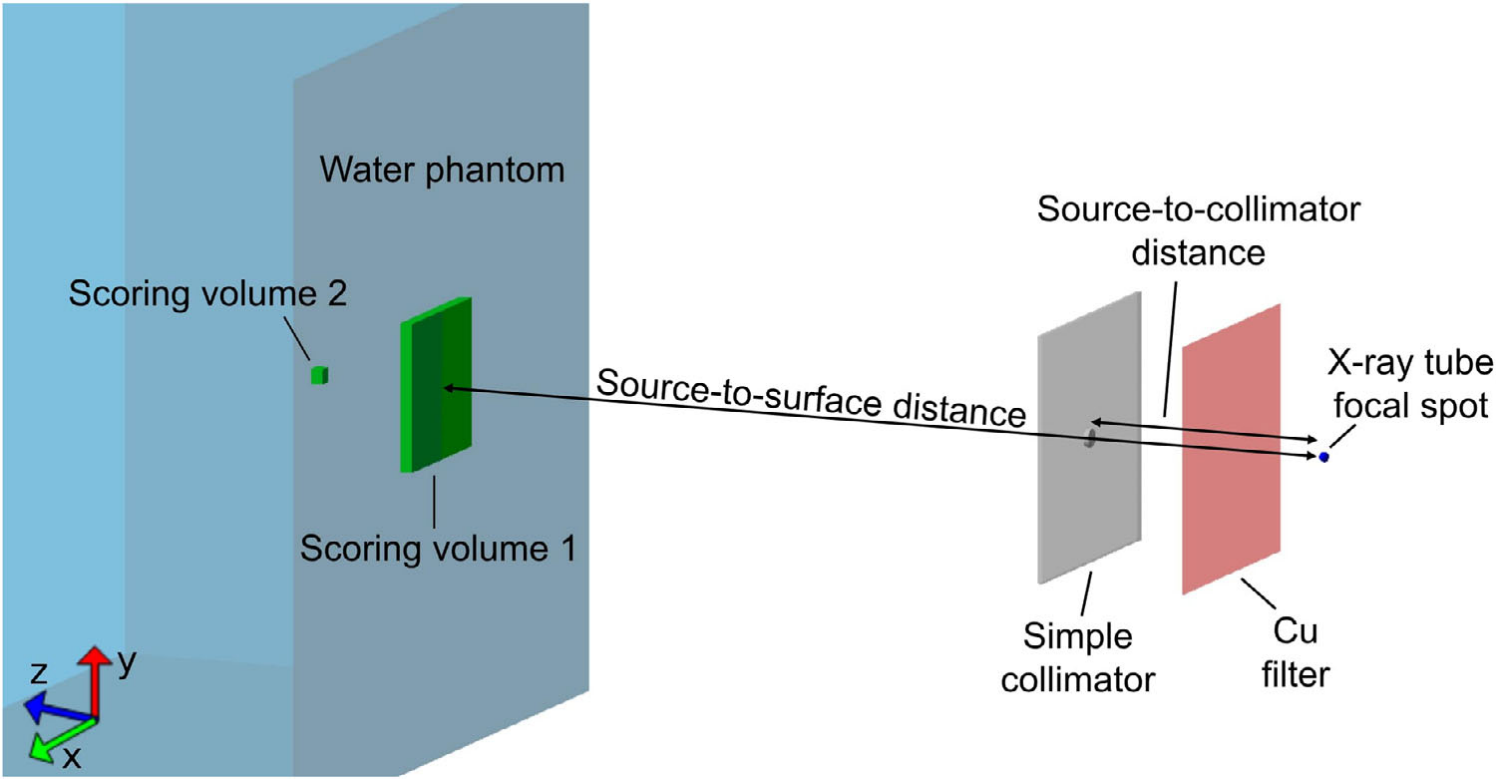
The MC simulation setup for Bayesian optimization iterations with indicated components. MC, Monte Carlo.

For these simulations, the beam passed through a copper filter at 1 cm from the phase space source, then through the simple collimator, and ultimately reached the water phantom encompassing scoring volume 1 (60×60×5) ${\text{mm}}^{3}$ on the phantom surface with (1×1×5) ${\text{mm}}^{3}$ voxels and scoring volume 2(3×3×5) ${\text{mm}}^{3}$ (one voxel) at 5cm depth each of which scored the mean dose and standard deviation per particle. Figure 3 shows the MC setup for the optimization simulations.

The scored mean dose per particle was converted to a normalized dose rate by multiplying by the maximum tube current of 20 mA for the large focal spot and 2.6 mA for the small focal spot and normalizing to the CAX mean dose rate for the existing system using the large focal spot. For each iteration of the optimizer, the surface dose rate was evaluated as the CAX mean dose rate of a (3 × 3) ${\text{mm}}^{2}$ region of interest (ROI) from scoring volume 1. The beam penumbra width was defined as the average of the penumbra widths in the x and y directions, which were determined by interpolating the 80% and 20% dose rate points. The OFD was calculated as the average dose rate within a 6 mm wide band along the outer edge of the 2D dose profile in both the x and y directions divided by the CAX dose rate. The dose at depth was considered as the mean dose rate in scoring volume 2 relative to the mean dose rate at the surface from the (3 × 3) ${\text{mm}}^{2}$ ROI of scoring volume 1.

The objective function to be minimized was defined as:

(1) $$\begin{array}{cc} F(x) & =\frac{{\dot{D}}_{\text{iris}}}{{\dot{D}}_{\mathrm{opt}}}w_{1}+\frac{P_{\mathrm{opt}}}{P_{\text{iris}}}w_{2}+\frac{OFD_{\mathrm{opt}}}{{\mathrm{OFD}}_{\text{iris}}}w_{3}+\frac{{\left({\mathrm{PDD}}_{\text{5cm}}\right)}_{iris}}{{\left(PDD_{5\mathrm{cm}}\right)}_{opt}}w_{4}-1,\\ w_{1} & =w_{2}=w_{3}=\frac{1}{6},\;w_{4}=\frac{3}{6}\;\text{for normalization,} \end{array}$$

where $\dot{D}$ is the surface dose rate, P is the mean beam penumbra width, OFD is the out‐of‐field dose, and ${\text{PDD}}_{5cm}=\frac{\dot{D}\left(5cm\right)}{\dot{D}\left(0cm\right)}$. The italic terms with a subscript *opt* are the terms calculated for each iteration of the optimizer, whereas the non‐italicized terms are the constant terms from the simulations of the current beam configuration as described in Section 2.3. The −1 was included so that negative objective function values indicated an improvement and positive objective function values indicated worsening. The depth dose term was weighted greater than the other terms to maximize the potential to treat deep‐seated targets, a primary goal of the system.

One hundred iterations of the Bayesian optimizer were run, each iteration taking approximately 35 min for a total optimization time of approximately 2 days and 10 h. This was done for both the large and small focal spots.

### 2.5 MC simulations of the optimized collimator and filtration

The optimal collimator and filtration parameters were selected based on the optimization results from the previous section. Simulations with a new iris collimator .stl file were then run with these optimal parameters identically to Section 2.3, only the leaflet thickness, copper filter thickness, SCD, and SSD were changed based on the optimization results (refer to Figure 2 for a visualization of the setup).

## 3 RESULTS

### 3.1 MC simulations of the current collimator and filtration

**FIGURE 4:**
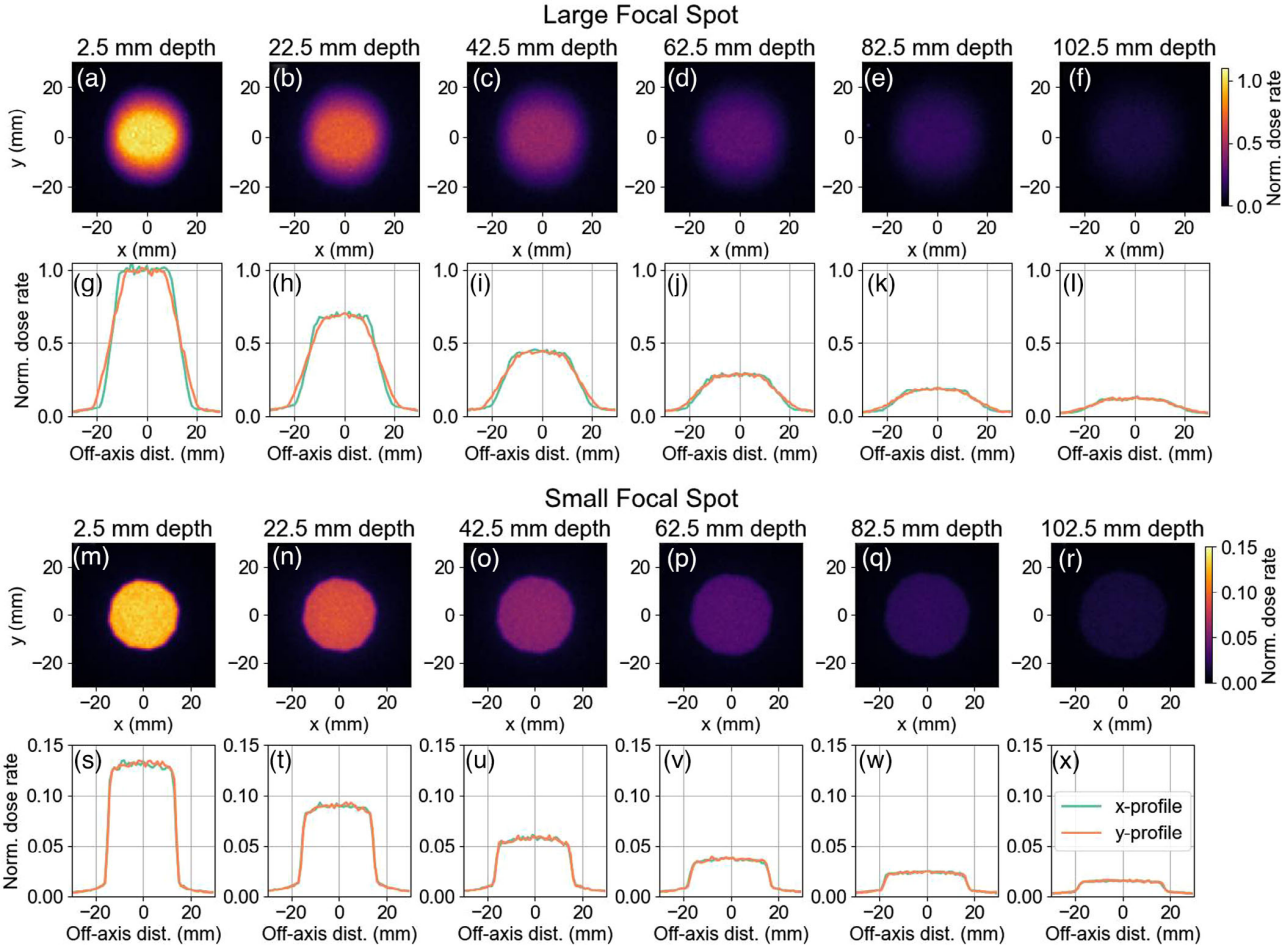
Simulated dose rate profiles from the existing iris collimator at depths in water ranging from 2.5 mm to 102.5 mm. Plots in (a–f) and (m‐r) represent 2D profiles, while (g–l) and (s‐x) show the corresponding 1D profiles.

2D and 1D dose rate profiles with depth for the existing beam configuration are shown in Figure 4.

The beam parameter values to be optimized are shown in Table 2 for the large and small focal spot sizes with the existing collimation and filtration configuration.

**TABLE 2:** Normalized CAX dose rate, mean beam penumbra width, OFD at surface, and ${\text{PDD}}_{5cm}$) for large and small focal spot sizes with the existing collimation and filtration configuration.

Focal spot size	Normalized CAX dose rate	Mean beam penumbra width (mm)	OFD (%)	${\text{PDD}}_{5cm}$
Large	(1.00±0.03)	(6.3±0.4)	(2.5±0.7)	(0.37±0.01)
Small	(0.131±0.005)	(2.2±0.4)	(2.5±0.6)	(0.37±0.01)

Abbreviations: CAX, central axis; OFD, out‐of‐field dose.

### 3.2 Bayesian optimization of beam collimation and filtration

**FIGURE 5:**
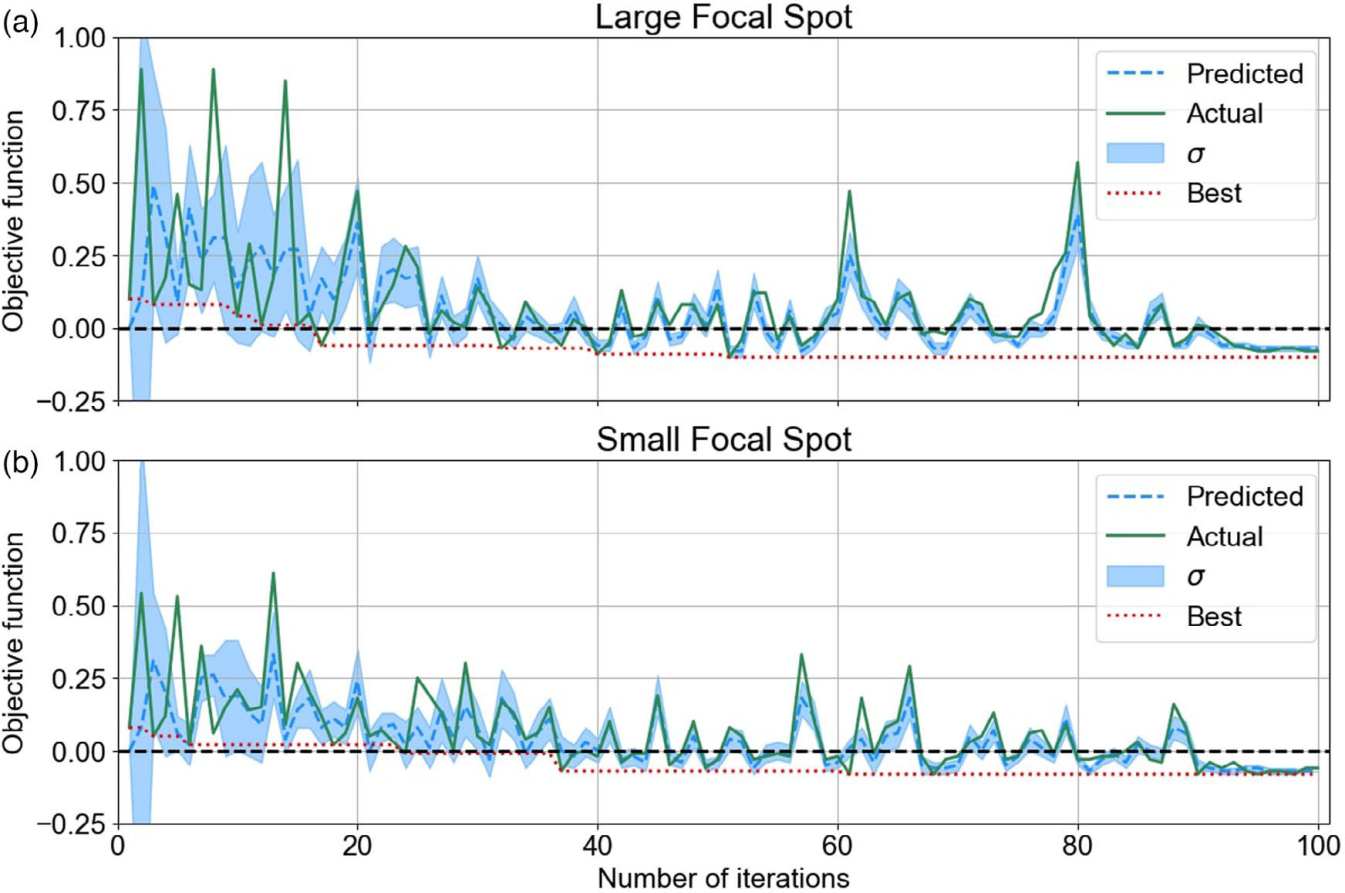
The actual objective function value and the Gaussian process predicted objective function values for each iteration for (a) the large and (b) the small focal spot.

The collimator and filtration optimization predicted and actual objective function values as a function of iteration number are shown in Figure 5. For both focal spot sizes, the agreement between the predicted and actual objective function values improved with the number of iterations. The minimum values for the objective function were −0.10 and −0.08 for the large and small focal spot sizes, respectively, indicating improvement from the existing configuration.

**FIGURE 6:**
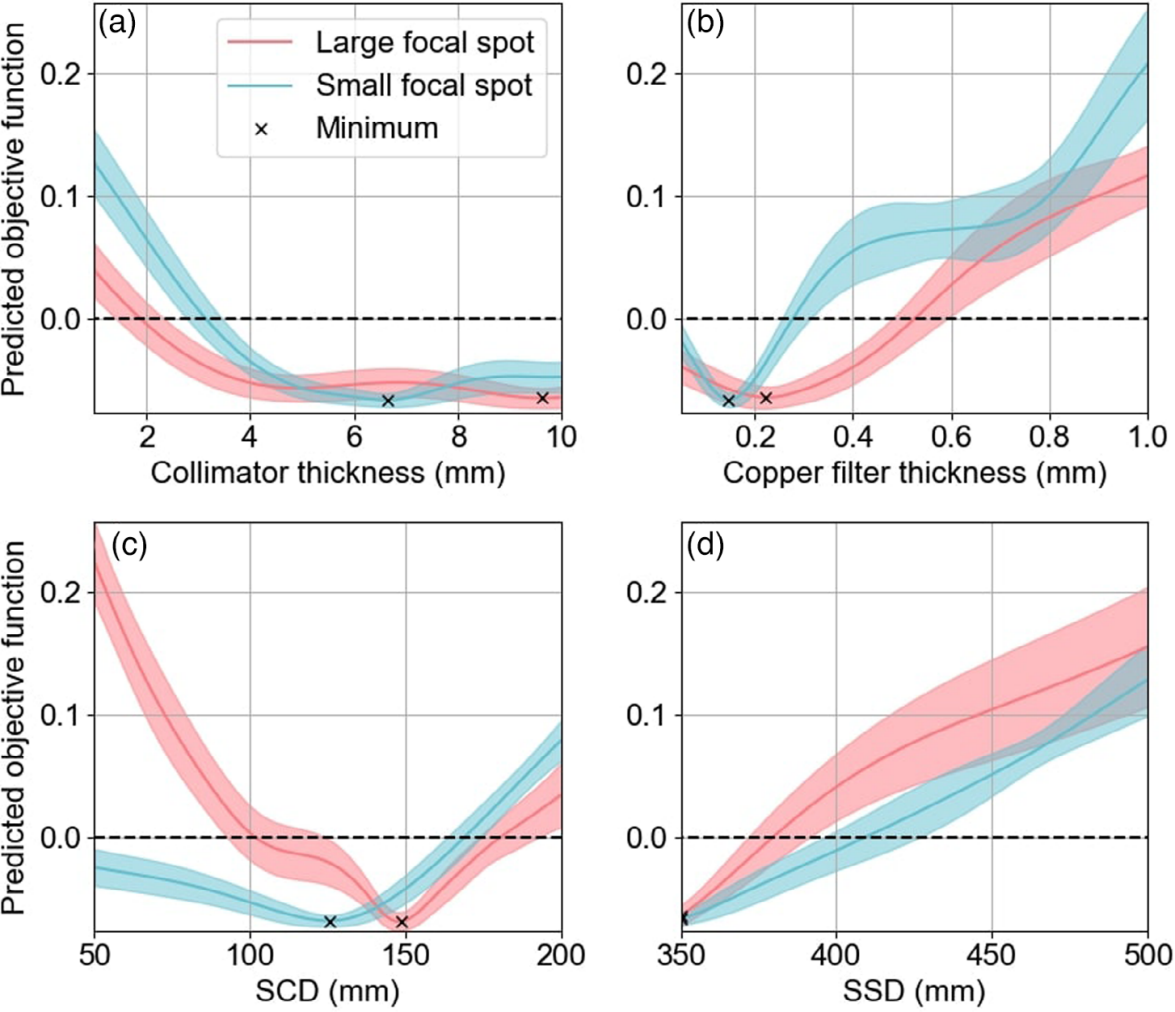
Gaussian process objective function predicted values for each variable. These plots show the predicted effect of changing one parameter while every other parameter is held at its optimal value.

The predicted objective function for each optimization variable over their considered range is shown in Figure 6. These plots show the predicted effect of changing one parameter at a time while every other parameter is at its optimal value.

Figure 6a shows the objective function decreasing as collimator thickness increased, before stabilizing with some variability at approximately 4 mm and 5 mm for the large and small focal spots, respectively. The collimator thicknesses that minimized the objective function were 9.6 mm for the large focal spot and 6.6 mm for the small focal spot. In Figure 6b, the copper filter thickness optimization yielded optimal values of 0.22 mm for the large focal spot and 0.15 mm for the small focal spot. Figure 6c displays the optimal SCD values, which were determined to be 148.5 mm for the large focal spot and 125.8 mm for the small focal spot. For SSD optimization, the best result for both focal spot sizes was the lower limit of the range considered, 350 mm.

### 3.3 MC simulations of optimized beam collimation and filtration

**FIGURE 7:**
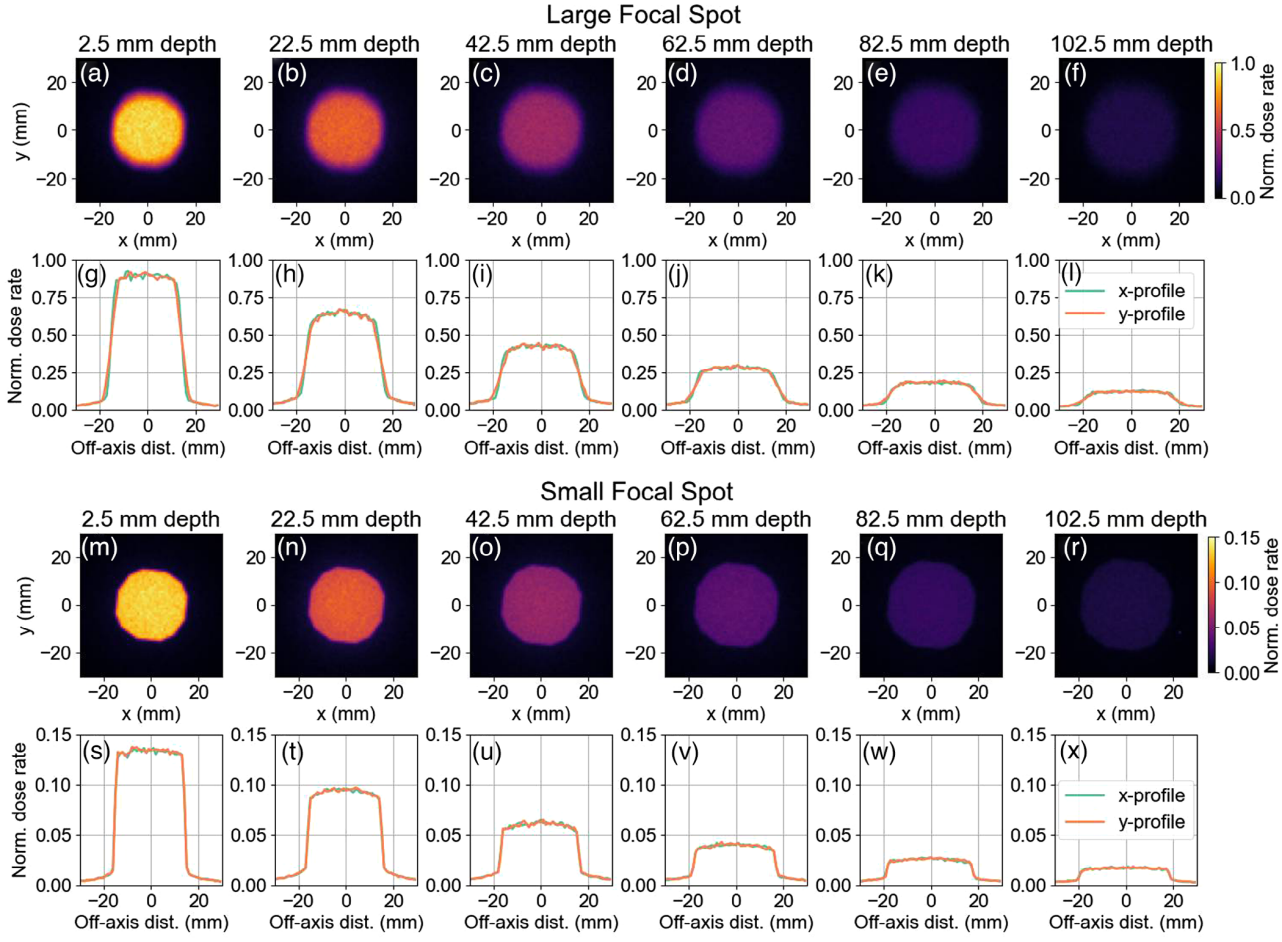
Simulated dose rate profiles for the optimized iris collimator and filtration at depths ranging from 2.5 mm to 102.5 mm in water. Plots in (a–f) and (m‐r) represent 2D profiles, while (g–l) and (s‐x) show the corresponding 1D profiles.

2D and 1D dose rate profiles with depth for the optimized iris collimator and beam configuration are shown in Figure 7.

**FIGURE 8:**
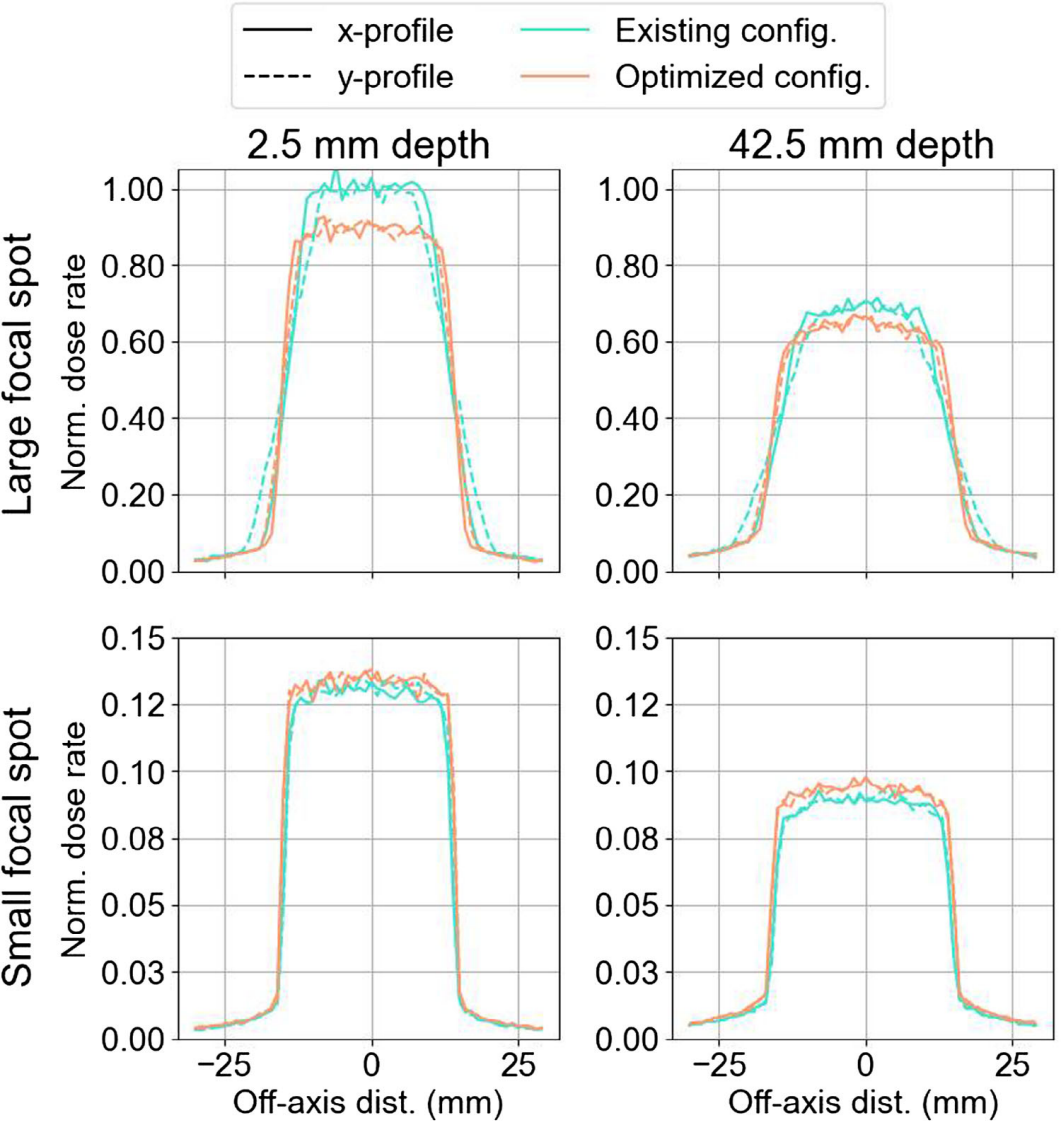
Normalized 1D dose rate profiles in x and y for the existing collimation and filtration configuration and the optimized configuration.

1D dose rate profiles comparing the existing collimation and filtration configuration with the optimized configuration are shown in Figure 8.

**FIGURE 9:**
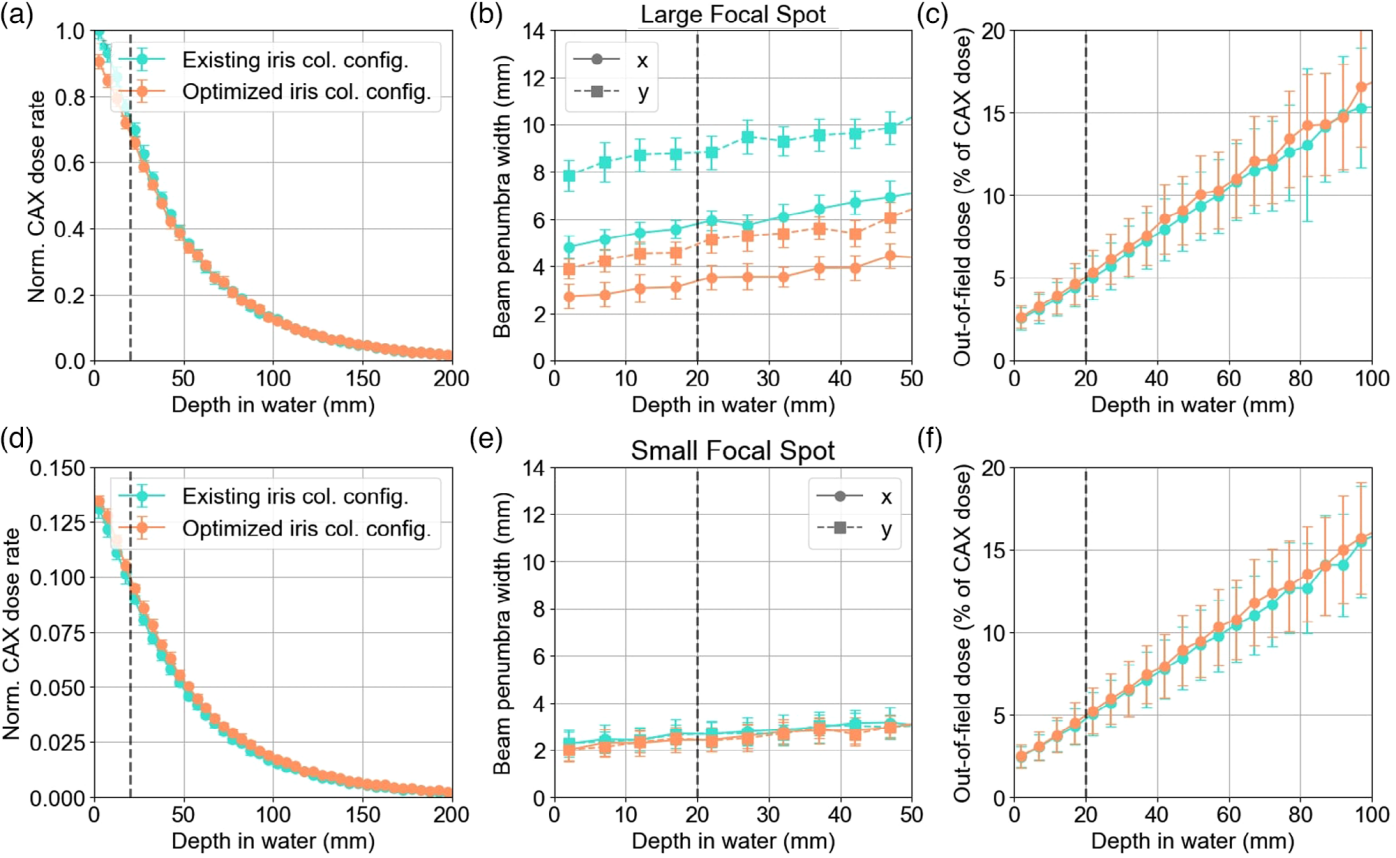
CAX dose rate, beam penumbra width, and OFD with depth in water for the existing iris collimator and filtration and the optimized iris collimator and filtration for both the large and small x‐ray tube focal spot sizes. A vertical dashed line indicates the isocenter at 20mm. CAX, central axis; OFD, out‐of‐field dose.

Figure 9 shows a comparison of beam properties calculated for the existing and optimized collimator and filtration. Specifically, CAX dose rate, beam penumbra width, and OFD as a function of depth in water are compared.

The beam parameter values of the objective function are shown in Table 3 for each focal spot size with the optimized collimation and filtration configurations.

**TABLE 3:** Normalized CAX dose rate, mean beam penumbra width, OFD at surface, and ${\text{PDD}}_{5cm}$) for large and small focal spot sizes with the optimized collimation and filtration.

Focal spot size	Normalized CAX dose rate	Mean beam penumbra width (mm)	OFD (%)	${\text{PDD}}_{5cm}$
Large	(0.91±0.02)	(3.3±0.3)	(2.6±0.7)	(0.40±0.01)
Small	(0.135±0.002)	(2.0±0.3)	(2.5±0.7)	(0.39±0.01)

Abbreviations: CAX, central axis; OFD, out‐of‐field dose.

For the large focal spot, the surface dose rate decreased by 9.4% for the optimized configuration, while the ${\text{PDD}}_{5cm}$ increased by 7.7%. Additionally, the surface beam penumbra width was reduced by 31.3%, and no significant changes in the OFD were observed within the margin of uncertainty. For the small focal spot, the surface dose rate increased by 3.7% and the ${\text{PDD}}_{5cm}$ increased by 5.3%, with no statistically significant changes in the beam penumbra width or OFD.

## 4 DISCUSSION

This study simulates the existing iris collimator and filtration of the KOALA RT system, performs a Bayesian optimization using TopasOpt, and ultimately proposes optimal configurations for both focal spot sizes of the x‐ray tube source.

To begin this work, the existing iris collimator and filtration were simulated using TOPAS MC. Figure 4 showcases 1D and 2D dose rate profiles for the large and small focal spots of the x‐ray source, the large focal spot had a greater CAX dose rate at each depth, however, the beam penumbra was significantly sharper for the small focal spot. Beam penumbra width and OFD (as a percent of CAX dose) were not significantly different between the two focal spot sizes. For the large focal spot, an asymmetry in beam size and penumbra is seen between the x and y axes which was a direct result of the large focal spot being significantly asymmetric.

For both focal spot sizes, the agreement between the predicted and actual objective function values improved with the number of iterations (see Figure 5). This indicates that the Bayesian optimizer accurately modeled the objective function as it collected more data points from the true objective function.

The optimal values for each collimation and filtration variable were then selected based on Figure 6. Since the objective function seemed to plateau with minimal fluctuation for a 5 mm or thicker collimator for both the large and small focal spot (see Figure 6), a 5 mm thick collimator was deemed optimal since it would be cheaper, lighter and with minimal loss in performance. Since the real collimator is an iris collimator and the x‐ray beam often passes through at least two leaflets, a 2.5 mm leaflet thickness was chosen as the optimal value. For the copper filter thickness and the SCD, the optimal value for each parameter was selected as the value at which a minimum of the objective function existed, these corresponded to interior minima with low uncertainty for both focal spots. In Figure 6d, the optimal SSD value was seen to be the minimum of the considered range, suggesting that minimizing SSD to as low as possible was optimal. Physically, a shorter SSD increases the CAX dose rate due to the inverse square law, reduces the beam penumbra width due to reduced beam divergence, and lowers the PDD due to the higher impact of inverse square law. This indicated that the improvement a shorter SSD offered to the dose rate and beam penumbra outweighed an expected loss in ${\text{PDD}}_{5cm}$ even with ${\text{PDD}}_{5cm}$ weighted three times greater in the objective function.

Figures 4 and 7 show beam profiles for the current and optimized configurations. The sharpness of the beam penumbra is noticeably improved for the optimized case. The asymmetry in beam size and penumbra also appears to be lessened, likely due to the shorter SSD lessening the effect of the focal spot size.

Several notable effects can be observed between the existing and the optimized configurations for the large focal spot. The 9.4% reduction in surface dose rate observed with the optimized collimator can be attributed to the thicker 0.22 mm copper filter, which more effectively attenuates the x‐ray beam compared to 0.1 mm copper. The 7.7% improvement in the ${\text{PDD}}_{5cm}$ can also be linked to the increased copper filtration, which increases PDD at greater depths. The substantial 31.3% decrease in beam penumbra width is due to several factors: primarily due to the increased SCD, which minimizes beam penumbra after collimation; the decreased SSD, which limits beam divergence before reaching the collimator; and the thicker collimator leaflets, which reduce collimator transmission. Despite the increased collimator thickness, the OFD remained the same suggesting that OFD is most likely dominated by scatter between the collimator and the phantom rather than leakage dose.

For the small focal spot, the surface dose rate increased slightly by 3.7%, which can be attributed to the decreased SSD despite the slightly thicker 0.15 mm copper filter. The ${\text{PDD}}_{5cm}$ increased by 5.3% due to the thicker copper filter. Unlike the large focal spot, the beam penumbra width did not improve significantly for the small focal spot, possibly because the beam penumbra was inherently narrower and less responsive to changes in optimization variables. Similar to the large focal spot, the OFD did not show significant changes with the thicker collimator leaflets, potentially also due to differences in overlap configuration resulting from the increased SCD.

Copper was the only filtration material considered in this study, as was done in previous work investigating kilovoltage radiotherapy.^23^ However, other filter materials, such as Sn and Al, present in Thoraeus filters, could be considered in future studies. Although the results are not presented, several other objective function weights were tested. The presented weights for the objective function terms were chosen because they yielded results and trends in the dosimetric parameters that agreed with the underlying physical principles expected when the geometric parameters were varied. To justify the choice of leaflet thicknesses of the iris collimator, which were based on the optimization that employed a simple block collimator, the dose rate, beam penumbra width, and OFD as a function of depth are compared for the optimized simple collimator and the optimized iris collimator (using STL files) for the large focal spot (Figure S1). An important next step for the KOALA system is the experimental validation of its MC model, which is the focus of a manuscript currently in preparation.

## 5 CONCLUSIONS

The optimal beam collimation and filtration for the dual‐robot KOALA system was determined using Bayesian optimization and MC simulations. This process refined key parameters, including iris collimator thickness, copper filtration, SCD, and sSSD, leading to a more effective treatment setup. Notably, for the studied values, collimator thickness did not significantly impact the OFD, and minimizing the SSD within system constraints was found to improve both dose rate and beam sharpness. Based on these findings, due to its sharper beam penumbra, treatments for which target dose conformity is paramount should be performed with the small focal spot. However, if treatment time is of great importance, treatments should be performed with the large focal spot due to the higher possible x‐ray tube power and achievable dose rate. The presented beam optimization enhances the KOALA system's therapeutic potential and improves treatment delivery for both focal spot sizes. Ultimately, the KOALA system will utilize non‐coplanar treatment delivery to achieve clinical doses to various deep‐seated tumor sites. Given the low penetration of kV x‐rays and the limitations of non‐coplanar arcs, the primary viable treatment sites will be lung, breast, and head and neck tumors.

## CONFLICT OF INTEREST STATEMENT

The authors declare no conflicts of interest.

## Supporting information

Supporting Information
