# Supplementary figures and images for: Beam collimation and filtration optimization for a novel orthovoltage radiotherapy system
Source: Med Phys. 2025 Feb 6;52(5):3204–15. doi: 10.1002/mp.17662 (PMC12059544; doi:10.1002/mp.17662)

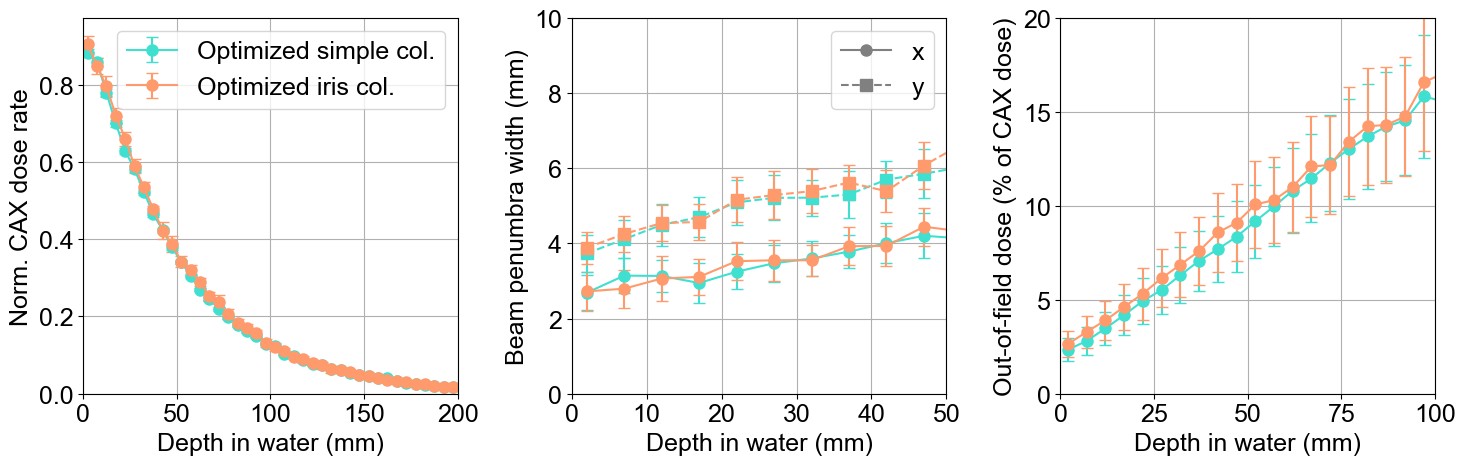

Supplement: Supplementary file 1 — Supporting Information [file MP-52-3204-s001.jpg]
